# The *PLIN4* Variant rs8887 Modulates Obesity Related Phenotypes in Humans through Creation of a Novel miR-522 Seed Site

**DOI:** 10.1371/journal.pone.0017944

**Published:** 2011-04-20

**Authors:** Kris Richardson, Qiong Louie-Gao, Donna K. Arnett, Laurence D. Parnell, Chao-Qiang Lai, Alberto Davalos, Caroline S. Fox, Serkalem Demissie, L. Adrienne Cupples, Carlos Fernandez-Hernando, Jose M. Ordovas

**Affiliations:** 1 Nutrition and Genomics Laboratory, Jean Mayer United States Department of Agriculture Human Nutrition Research Center on Aging at Tufts University, Boston, Massachusetts, United States of America; 2 Department of Biostatistics, Boston University School of Public Health/Boston University Medical Campus, Boston, Massachusetts, United States of America; 3 Department of Epidemiology, School of Public Health, and Clinical Nutrition Research Center, University of Alabama at Birmingham, Birmingham, Alabama, United States of America; 4 Departments of Medicine and Cell Biology, Leon H. Charney Division of Cardiology and the Marc and Ruti Bell Program in Vascular Biology and Disease Program, New York University School of Medicine, New York, New York, United States of America; 5 Framingham Heart Study and the Center for Population Studies, National Heart, Lung, and Blood Institute, Framingham, Massachusetts, United States of America; 6 National Heart, Lung, and Blood Institute, Framingham Heart Study, Framingham, Massachusetts, United States of America; 7 Instituto Madrileño de Estudios Avanzados (IMDEA) Alimentacion, Madrid, Spain; 8 Department of Cardiovascular Epidemiology and Population Genetics, Centro Nacional de Investigaciones Cardiovasculares (CNIC), Madrid, Spain; Aarhus University, Denmark

## Abstract

*PLIN4* is a member of the PAT family of lipid storage droplet (LSD) proteins. Associations between seven single nucleotide polymorphisms (SNPs) at human *PLIN4* with obesity related phenotypes were investigated using meta-analysis followed by a determination if these phenotypes are modulated by interactions between *PLIN4* SNPs and dietary PUFA. Samples consisted of subjects from two populations of European ancestry. We demonstrated association of rs8887 with anthropometrics. Meta-analysis demonstrated significant interactions between the rs8887 minor allele with PUFA n3 modulating anthropometrics. rs884164 showed interaction with both n3 and n6 PUFA modulating anthropometric and lipid phenotypes. *In silico* analysis of the *PLIN4* 3′UTR sequence surrounding the rs8887 minor A allele predicted a seed site for the human microRNA-522 (miR-522), suggesting a functional mechanism. Our data showed that a PLIN4 3′UTR luciferase reporter carrying the A allele of rs8887 was reduced in response to miR-522 mimics compared to the G allele. These results suggest variation at the *PLIN4* locus, and its interaction with PUFA as a modulator of obesity related phenotypes, acts in part through creation of a miR-522 regulatory site.

## Introduction

The World Health Organization (WHO) estimates 1.6 billion people are overweight, and 400 million obese (www.who.int). Those affected are at increased risk for occurrence of cardiovascular diseases (CVD) and other chronic conditions that reduce both quality of life and life expectancy [1], [2]. The basis for obesity is the inability of the individual to maintain the balance between energy uptake, storage and expenditure. Adipose tissue plays a critical role in this complex equilibrium, as well as protecting against the potential lipo-toxic damage of circulating free fatty acids (FFAs) by acting as an intracellular sink for triacylglycerols (TAG) in lipid storage droplets (LSDs) [3]. It has been hypothesized that some of the adverse metabolic consequences related to obesity are the result of saturating the buffering capacities of the adipose tissue resulting in an overflow of FFAs toward other non-adipose tissues, a process which has been associated with insulin resistance and decreased clearance of TAG rich particles [4].

PLIN4 is a member of the PAT family of LSD proteins, also known as the Perilipins PLIN1/*perilipin (* ***P*** *LIN)*, *PLIN2/adipose differentiation related protein (* ***A*** *DRP)*, *PLIN3/tail interacting protein 47 (* ***T*** *IP47)*, *PLIN4/S3-12 and PLIN5/Lipid Storage Droplet Protein 5 (LSDP5)* [5]. PLIN4 is expressed mainly in adipose and relocates to forming LSDs from a scattered distribution in the cytoplasm of 3T3-L1 cells when stimulated with insulin and oleate. Upon their removal, PLIN4 returns to its basal state location in the cell periphery suggesting PLIN4 facilitates uptake of FFAs from the blood to the LSD in response to the nutritional state of the cell [6]. Importantly, several *in vivo* and *in vitro* studies support the relevant role of PLIN1, and the other PATs, in the regulation of LSD TAG stores [6], [7], [8].

It has been proposed that common complex diseases occur as a consequence of common genetic variation - the common disease, common variant hypothesis [9]. In this scenario risk for disease is dependent on the collective contribution of genetic variants, with small to moderate effect size, which an individual may carry [9]. Indeed, candidate-gene and genome-wide association studies have identified numerous SNPs influencing obesity risk but many of these associations have not been replicated, partly due to weak experimental design and to potential interactions between multiple genetic and non-genetic factors, such as diet [10], [11], [12]. Thus, the need to investigate these interactions to define more precisely, both an individual's disease risk and the most appropriate therapeutic approach.

The promoter regions of *PLIN1* and *PLIN4* contain conserved and functional peroxisome proliferator-activated receptor (PPAR) response-elements (PPREs) [8]. PPARs are a family of nuclear-receptor transcription factors that modulate many aspects of lipid metabolism [13]. Polyunsaturated fatty acids (PUFA) are known ligands for PPAR receptors suggesting PAT genes respond to dietary lipids at the transcriptional level. Interestingly, several human studies showed that genetic variation at the *PLIN1* locus associates with anthropometric phenotypes in female subjects [14], [15], [16]. Moreover, other studies have demonstrated gene by environment interactions for *PLIN1* influencing weight in response to Rosiglitazone and insulin resistance levels for women consuming diets high in saturated fat [17], [18].

Although there have been numerous functional investigations into variants located in the promoter regions of candidate genes little attention has been given to variants falling in the 3′UTR where microRNAs (miR)s may bind. miRs regulate protein output, and individual miR-to-target mRNA interactions may act to dampen mRNA translation often by 33% or less [19]. In line with the common disease-common variant hypothesis it has been proposed that variants mapping within miR targets, or which create novel miR-to-target interactions, have functional consequence resulting in subtle phenotypic variation [20].

We hypothesize that variation in human *PLIN4* may modulate obesity related phenotypes. To explore this, we performed a sample size weighted meta-analysis using results from association analysis of seven *PLIN4* SNPs with anthropometric, lipid and glucose variables in two populations, the Genetics of Lipid Lowering Drugs and Diet Network (GOLDN) and the Framingham Offspring Study (FOS). We also investigated the interaction of dietary PUFA n3 and n6 with *PLIN4* SNPs to determine their combined potential to modulate these phenotypes. *In silico* prediction for SNPs falling in *PLIN4* regulatory regions was done to assess their potential for functional consequence. Our results indicated the rs8887 SNP creates a miR-522 miR recognition element (MRE) in the *PLIN4* 3′UTR. The ability of miR-522 to regulate *PLIN4* 3′UTR was examined. We investigated how genetic drift in the *PLIN4* 3′UTR in combination with environmental exposures may act in concordance, predisposing individuals to obesity. Although our association results were not adjusted for multiple tests, the combined evidence of meta-analysis and functional *ex-vivo* data, indicates rs8887 as a modulator of anthropometrics in humans.

## Results

### Meta-analysis of *PLIN4* variants with anthropometric, lipid and glucose related phenotypes from the FOS and GOLDN populations

**Figure 1:**
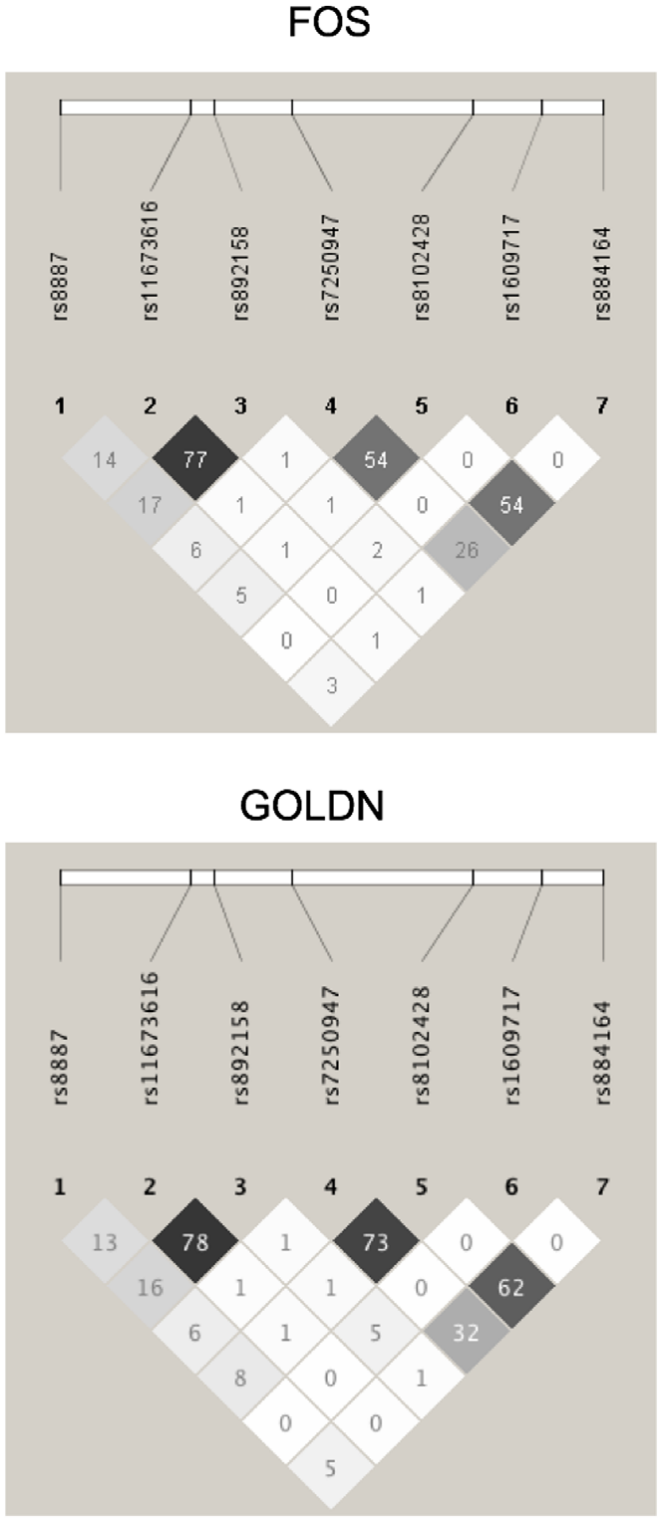
LD Plot of *PLIN4* SNPs in FOS & GOLDN. LD plots were generated in the Haploview program using unrelated individuals from the corresponding studies. The r^2^ LD estimate was used for both populations and is reported in the figure above.

The demographic and biochemical characteristics of participants for the FOS and GOLDN populations varied slightly, and are presented in **Table 1**. Genotypic characteristics are shown in **Table 2**. Genotype distributions did not deviate from Hardy-Weinberg equilibrium. Linkage disequilibrium (LD) between SNPs varied slightly between populations (**Figure 1**).

**Table 1:** Demographic and biochemical characteristics of FOS & GOLDN subjects.

	Men		Women	
	FOS (N = 1259)	GOLDN (N = 481)	FOS (N = 1352)	G0LDN (N = 513)
**Trait**	Mean (SD)	Mean (SD)	Mean (SD)	Mean (SD)
**Age (years)**	56.3(9.9)	52.9(14.4)	55.9(9.6)	52.2(14.0)
BMI (kg/m**2)	28.5(4.16)	29(4.58)	27.1(5.51)	28.4(6.19)
**Waist (cm)**	100.3(10.6)	102.1(11.78)	90.42(14.07)	93.21(17.9)
**Weight (kg)**	87.49(14.09)	82.91(14.01)	70.59(15)	69.34(15.43)
**Waist/Hip Ratio**	0.97(0.05)	0.96(0.09)	0.87(0.08)	0.85(0.09)
**Glucose (mg.dL)**	106(26.7)	106.4(20.5)	98.7(23.6)	98.6(16.3)
**Homa** *	8.7(7.09)	3.85(2.86)	7.26(5.93)	3.35(2.51)
**Insulin (mU/L)**	3.41(0.37)	14.21(8.60)	3.3(0.32)	13.2(8.08)
**Triglycerides (mg/dL)**	156(108)	150.7(92.9)	133(80.8)	129(80.1)
**HDL Cholesterol (mg/dL)**	43.2(10.9)	41.5(10)	56.9(14.6)	52.9(14)
**Total PUFA n3 - g**	1.43(0.58)	1.83(0.98)	1.37(0.53)	1.48(0.82)
**Total PUFA n6 - g**	11.03(5.12)	18.2(10.23)	9.79(4.37)	14.1(7.87)
**Food Energy - kcal**	1989(630)	2354(896)	1735(546)	1719(629)
**Physical Activity Score**	-	35.2(7.39)	-	33.1(4.73)

	%Use	%Use	%Use	%Use
**Current Smokers (%)**	16	8	18	8
**Current Alcohol Users (%)**	73	51	60	52
**Menopausal (%)**	-	-	69	-
**Hormones (%)**	-	-	23	20
**Taking Hypertensive Medications (%)**	12	23	9	17
**Taking Diabetes Medications (%)**	12	5	7	4
**Taking Cholesterol Medications (%)**	12	6	8	4

Data are means and standard deviation (SD) for continuous variables or % usage for categorical variables. Populations are displayed by gender for all anthropometric, lipid and glucose variables investigated. The percent usage of tobacco, alcohol, hormone, hypertension, diabetes and cholesterol medication is also listed. The % of menopausal women is provided for FOS, only.

*Homeostasis model assessment of insulin resistance (homa).

**Table 2:** Genotypic characteristics of *PLIN4* SNPs in FOS & GOLDN subjects.

*PLIN4*				FOS		GOLDN	
SNP	Allele	Position	Feature	Minor	HWE-P	Minor	HWE-P
rs8887	G/A	4453201	3′UTR	0.45	0.92	0.45	0.81
rs11673616	A/G	4457915	Intronic	0.13	0.68	0.11	0.42
rs892158	G/A	4458716	Intronic	0.16	0.38	0.14	0.91
rs7250947	G/A	4461530	Exonic	0.09	0.40	0.07	0.43
rs8102428	A/G	4467982	Intronic	0.10	0.80	0.09	0.49
rs1609717	T/C	4470450	Promoter	0.05	0.05	0.06	0.80
rs884164	T/C	4472625	Promoter	0.08	0.61	0.07	0.03

*PLIN4* is found on Chromosome 19. dbSNP rs numbers for each SNP genotyped are given in column one. Major and minor alleles, and chromosomal position (GRCh 36.3) are provided, followed by the gene region in which the SNP falls. Allele frequencies and Hardy-Weinberg equilibrium p-values are given for each SNP in FOS and GOLDN populations.

To test for overall significance of association of *PLIN4* SNPs with phenotypes of interest, we performed a meta-analysis that revealed significant associations between rs8887 and BMI (*P = *0.002), weight (*P = *0.017) and a nominal association with waist circumference (*P = *0.056) with minor allele carriers having elevated measures in each case (**Table 3**). We report here only those associations for SNPs showing consistent trends with supporting functional hypotheses. A complete list of our findings can be viewed in **Table S1**. The direction of these effects is in agreement with those reported for the FOS and GOLDN populations. For our main effect analyses, variation at rs8887 explained 0.4% and 0.33% of variance of BMI in FOS and GOLDN, respectively.

**Table 3:** Significant Associations of *PLIN4* SNPs in the FOS and GOLDN populations - Main Effects.

			FOS				GOLDN				Meta-Analysis	
SNP	Phenotype	Gender	Beta	Se	P	%Var	Beta	Se	P	%Var	z-score	P
rs8887	BMI	Both	0.614	0.221	0.005	0.396	.581	0.378	0.125	0.334	3.164	0.002
		Males	0.624	0.269	0.021		0.326	0.461	0.480		2.319	0.020
		Females	0.631	0.335	.060		0.704	0.586	0.230		2.231	0.026
	Weight	Both	3.106	1.431	0.030	0.200	2.374	2.271	0.296	0.081	2.387	0.017
		Males	2.917	1.980	0.141		0.584	3.146	0.853		1.332	0.183
		Females	3.524	2.010	0.080		3.465	3.242	0.286		2.050	0.040
	Waist	Both	0.423	0.221	0.056	0.157	0.252	0.440	0.567	0.023	1.911	0.056
		Males	0.483	0.269	0.073		−0.085	0.565	0.880		1.413	0.158
		Females	0.381	0.334	0.253		0.397	0.656	0.556		1.278	0.201
	VAT	Both	199.5	67.55	0.003							
		Males	345.4	100.5	0.001							
		Females	68.7	83.97	0.413							
	SAT	Both	229.1	90.66	0.011							
		Males	180.4	106.2	0.090							
		Females	248.9	147.2	0.054							

Results of meta-analysis in FOS and GOLDN performed using a Dominant Model, due in part to low allele frequencies of several SNPs. P-values for anthropometrics were adjusted for sex, age, smoking, physical activity (GOLDN only), alcohol use, diabetes, beta-blockers, calories from fat, PUFA n3 and n6, and estrogen and menopausal status (FOS only) in women. Lipid and glucose p-values were also adjusted for BMI and cholesterol medications.

### Meta-analysis of interaction of dietary n3 and n6 PUFA with *PLIN4* variants on anthropometric, lipid and glucose related phenotypes from the FOS and GOLDN populations

We performed a meta-analysis of interaction between *PLIN4* SNPs and PUFA n3 and n6. The interaction between PUFA n3 and rs8887 showed significant association modulating BMI (*P = *0.0144), weight (*P = *0.0068) and waist circumference (*P = *0.0145) where minor allele carriers showed reduced anthropometrics in response to PUFA n3 compared to non-carriers. An interaction between rs884164 and PUFA n3 showed BMI (*P = *0.008), weight (*P = *0.005), waist (0.035), glucose (*P = *0.0167) and TAG (*P = *0.0144) levels are increased in carriers of the minor allele with elevated PUFA n3 intake (**Table 4**). Furthermore, rs884164 showed significant interaction with PUFA n6 with HDL (P = 0.036) levels decreasing and TAG (P = 0.012) increasing among minor allele carriers with elevated PUFA n6 intake (**Table 4**).

**Table 4:** Significant *PLIN4* by diet interactions from meta-analysis.

				FOS				GOLDN				Meta-Analysis	
SNP	Phenotype	PUFA	Gender	Beta	Se	P	%Var	Beta	Se	P	%Var	z-score	P
**rs8887**	BMI	n3	Both	−0.469	0.391	0.230	0.48	−1.208	0.459	0.009	0.77	−2.447	0.014
			Males	−0.624	0.466	0.181		−1.158	0.542	0.033		−2.288	0.022
			Females	−0.438	0.625	0.484		−0.964	0.838	0.251		−1.216	0.224
	Weight	n3	Both	−3.867	2.522	0.125	0.30	−7.189		0.009	0.44	−2.707	0.007
			Males	−3.778	3.430	0.271		−7.080		0.057		−1.964	0.049
			Females	−4.553	3.750	0.225		−6.046		0.194		−1.728	0.084
	Waist	n3	Both	−0.461	0.391	0.238	0.23	−1.444	0.544	0.008	0.55	−2.445	0.015
			Males	−0.500	0.466	0.283		−1.230	0.672	0.068		−1.900	0.057
			Females	−0.421	0.621	0.498		−1.584	0.952	0.097		−1.483	0.138
**rs884164**	BMI	n3	Both	1.077	0.458	0.019	0.17	0.875	0.694	0.208	0.13	2.655	0.008
			Males	0.924	0.534	0.084		0.932	0.798	0.243		2.087	0.037
			Females	0.974	0.799	0.223		1.700	1.347	0.208		1.709	0.087
	Weight	n3	Both	6.860	2.995	0.020	0.12	6.689	4.116	0.109	0.12	2.819	0.005
			Males	6.202	3.958	0.117		8.806	5.443	0.106		2.195	0.028
			Females	5.475	4.801	0.254		7.803	7.490	0.298		1.524	0.128
	Waist	n3	Both	1.164	0.459	0.011	0.19	−0.015	0.791	0.985	0.01	2.106	0.035
			Males	0.938	0.538	0.081		−0.230	0.900	0.798		1.317	0.188
			Females	0.920	0.793	0.247		0.761	1.543	0.622		1.239	0.216
	Triglycerides	n3	Both	0.086	0.046	0.062	0.13	0.111	0.069	0.106	0.31	2.448	0.014
			Males	0.152	0.067	0.024	0.48	0.240	0.094	0.011	1.45	3.289	0.001
			Females	0.003	0.064	0.958		−0.073	0.116	0.527		−0.303	0.762
		n6	Both	0.014	0.006	0.011	0.24	0.005	0.007	0.479	0.11	2.503	0.012
			Males	0019	0.008	0.013	0.52	0.02	0.009	0.035	1.07	3.229	0.001
			Females	0.007	0.008	0.421		−0.012	0.011	0.274		0.073	0.942
	Glucose	n3	Both	0.006	0.012	0.620	0.03	0.056	0.016	0.001	0.90	2.393	0.017
			Males	0.009	0.016	0.578		0.082	0.023	0.001		2.379	0.017
			Females	0.005	0.018	0.788		0.021	0.024	0.398		0.689	0.491
	HDL	n6	Both	−0.207	0.135	0.125	0.03	−0.215	0.145	0.138	0.31	−2.096	0.036
			Males	−0.194	0.153	0.204	0.11	−0.342	0.163	0.036	0.71	−2.215	0.027
			Females	−0.086	0.252	0.734		−0.019	0.267	0.942		0–.324	0.746

Gene by diet interaction for meta-analysis of FOS and GOLDN. Interactions between *PLIN4* variants and dietary PUFA n3 and n6 were included in a multivariate regression model as continuous variables. Models were adjusted as in Table 3.

Performing the meta-analysis separately for each gender revealed several associations in the male population. Interactions were observed between rs884164 and PUFA n3 in which BMI (*P = *0.037), weight (*P = *0.028), glucose (*P* = 0.017) and TAG (*P = *0.001) levels were modulated. The minor allele subjects showed elevated levels for each trait, compared to non-carriers. The percent of variance was calculated for anthropometric traits explained by the interaction of *PLIN4* variants and PUFA intake. Variation at rs8887 and PUFA n3 intake explained 0.48% and 0.77% of variance in BMI in FOS and GOLDN, respectively. Variation at rs884164 and PUFA n3 intake explained 0.17% and 0.13% of variance in BMI in FOS and GOLDN, respectively (**Table 4**).

### Association of *PLIN4* variants with visceral and subcutaneous fat measurements from the FOS population

Visceral adipose tissue (VAT) has been shown to correlate better with obesity related phenotypes such as insulin resistance and CVD than the more traditional anthropometrics [21]. We performed an association analysis with *PLIN4* SNPs and volumetric computed tomography measures from a subset of the FOS study for whom those measures were taken. rs8887 associated with VAT (*P = *0.003) where carriers of the minor allele showing increased volume compared to non-carriers. In addition carriers of the rs8887 minor allele showed increased subcutaneous adipose tissue (SAT) (*P = *0.011) compared to non-carriers. Performing our analyses by gender revealed an association with rs8887 and VAT (*P = *0.00059) with only male carriers having greater volume than non-carriers (**Table 3**). Variation in rs8887 explained 0.75% of variance in VAT and 0.56% of variance in SAT in the FOS population.

### Functional prediction of *PLIN4* variants

**Figure 2:**
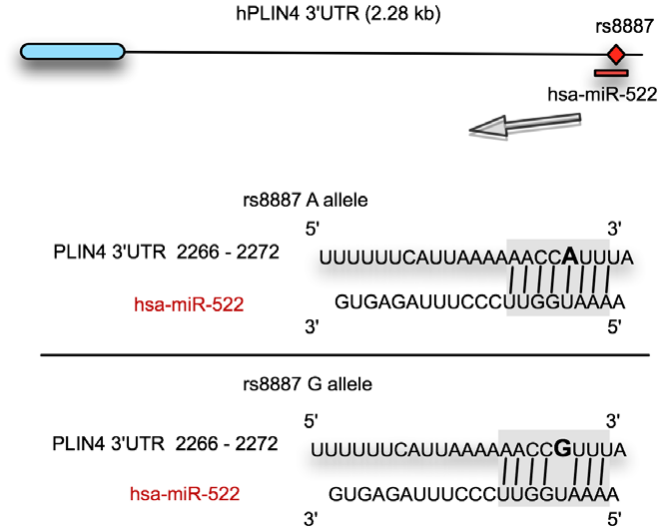
The rs8887 minor A allele creates a novel miR-522 MRE in the *PLIN4* 3′UTR. Diagram of the miR-522:*PLIN4* 3′UTR sequences with the A or G allele. The miR-522 seed site is highlighted in gray, and the rs8887 variants are in bold.

We investigated if variants showing the most consistent associations may be functional. Using CEU data from HapMap we determined that rs8887 was not in LD with other known SNPs. As rs8887 is located in the 3′UTR of the *PLIN4* mRNA, we searched for miRs predicted to bind the *PLIN4* mRNA using Targetscan.org and microRNA.org [22], [23]. Both programs predicted the binding of miR-522 with perfect complementarity to a seed site in the *PLIN4* mRNA when containing the minor A allele (**Figure 2**). In HapMap, rs884164 was estimated to be in LD with the downstream SNP rs8102428 (r^2^ = 0.90), and the upstream SNPs rs11670485 (r^2^ = 0.91) and rs892157 (r^2^ = 0.90). Our data showed that the LD between rs884164 and rs8102428 in GOLDN (r^2^ = 0.7) and FOS (r^2^ = 0.54) were weaker than predicted suggesting that use of HapMap values to estimate LD at this locus may not be ideal. rs884164 falls in the promoter region of *PLIN4* and sequence analysis using the transcription factor motif prediction tool Mapper indicated that the major T allele lies in a consensus NFkB motif and the C allele abrogates this prediction [24].

### miR-522 targets the 3′UTR of *PLIN4* containing the rs8887 minor A allele

**Figure 3:**
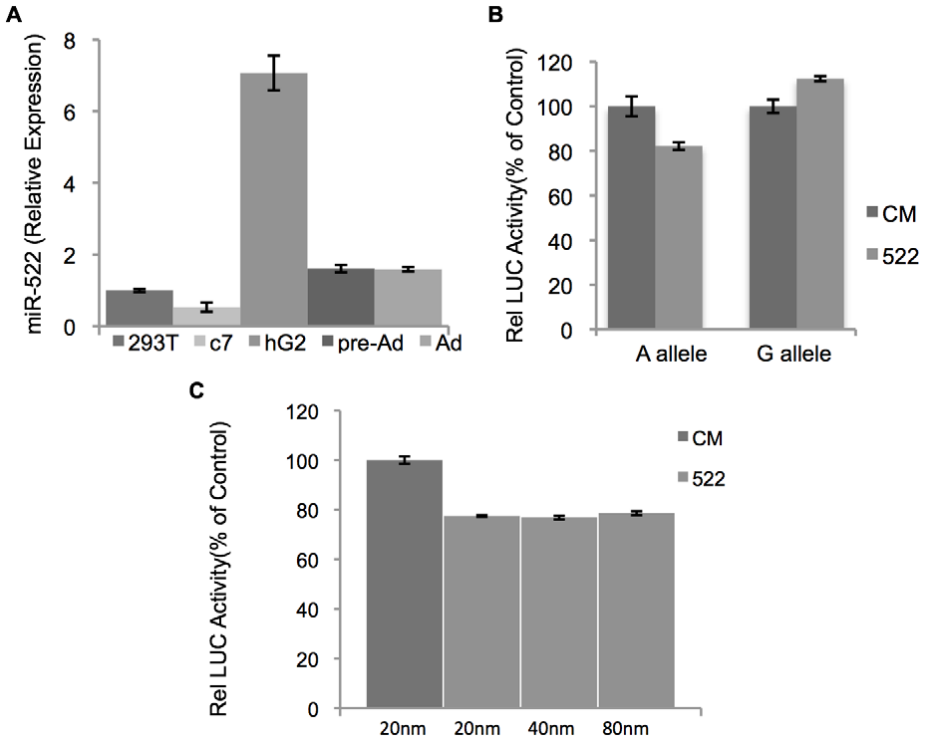
The *PLIN4* 3′UTR with the A allele creates a miR-522 MRE. A) Relative miR-522 expression across indicated cell types, hek293T (293T), Cos7 (c7), hepG2 (hG2), pre-adpocytes (pre-Ad) and adipocytes(Ad). B) Luciferase expression of pmiR-LucPLIN4-G or A constructs with miR-522 (522) or control mimic (CM). Data are expressed as relative luciferase activity to control samples. C) Luciferase expression of pmiR-LucPLIN4-A constructs with increasing concentration of miR-522 compared to control mimic. All data represent experiments performed in triplicate. Statistical Analysis: P values for the difference between luciferase activity obtained for LucPLIN4-A in the presence of mir-522 or control mimic (P = .0169) or LucPLIN4-G in the presence of mir-522 or control mimic (P = .0584) were determined using the student's paired t-test.

We next investigated the functional potential of rs8887. miR-522 maps within the chromosome 19 microRNA cluster (C19MC), the largest known primate specific microRNA gene cluster [25], [26]. While there is evidence for miR-522 expression in placenta, testis, thymus, brain and prostate to our knowledge expression has not been demonstrated in adipose tissue [25], [27]. Thus to determine if miR-522 is expressed in human adipoctyes, we performed RT-PCR on total RNA samples extracted from cultured COS7, HEK293T, HepG2 cells, and primary human pre-adipocyte and mature adipocytes. This was followed by qPCR using SABiosciences miR-522 specific primers. **Figure 3a** shows the normalized relative expression of miR-522, with highest expression in HepG2 cells, pre-adipocytes and adipocytes. *PLIN4* has been shown to be expressed in human adipose [28].

To determine the effect of miR-522 on the *PLIN4* 3′UTR, we cloned into the siCHECK2 luciferase expression vector a 560-bp region of the *PLIN4* 3′UTR from genomic DNA of subjects homozygous for either rs8887 allele. COS7 cells were co-transfected with miR-522 mimic or control mimic, and with the A allele or the G allele *PLIN4* 3′UTR vector. The 3′UTR containing the minor A allele showed 20% reduction in luciferase signal in the presence of miR-522 compared to control mimic (**Figure 3b**). The 3′UTR containing the G allele showed a non-significant increase in luciferase signal in the presence of miR-522 compared to control mimic. These data indicate an ability of miR-522 to bind and partially repress luciferase expression via the *PLIN4* 3′UTR segment when carrying the derived A allele of rs8887. The effect of increasing concentrations of miR-522 on PLIN4 3′UTR with the A allele is saturated at 20 nM of miR-522 (**Figure 3c**).

### Genetic drift and the *PLIN4* 3′UTR

**Figure 4:**
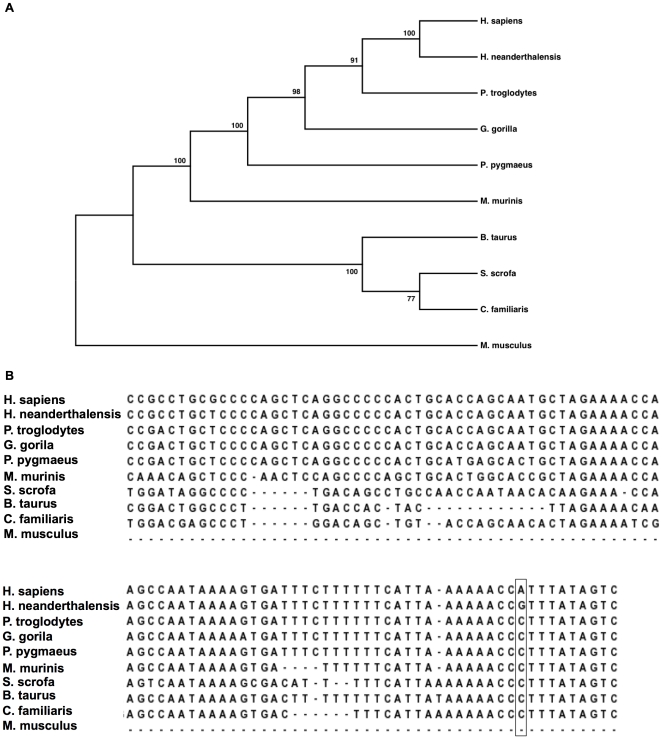
Evolutionary history of the *PLIN4* 3′UTR. A) The evolutionary history of *PLIN4* was inferred using the Maximum Parsimony method. The bootstrap consensus tree inferred from 10000 replicates is taken to represent the evolutionary history of the taxa analyzed. The percentage of replicate trees in which the associated taxa clustered together in the bootstrap test are shown next to the branches. The MP tree was obtained using the Close-Neighbor-Interchange algorithm with search level 3 in which the initial trees were obtained with the random addition of sequences (100 replicates). B) Clustal W was used to align the *PLIN4* 3′UTR sequences from H. sapiens, H. neanderthalensis, P. troglodytes, G. gorilla, P. pygmaeus, M. murinis, C. familiaris, B. Taurus, S. scrofa, and M. musculus. The last 100 bases of the alignment are show here, with the position harboring the rs8887 SNP outlined.

Genetic drift occurring in 3′UTRs can result in the formation of new MREs. These new MREs confer beneficial, neutral or detrimental effects on the organism, leading to conservation, neutrality or selective avoidance of the MRE, and such mechanisms have had considerable influence on 3′UTR evolution [19]. We examined the *PLIN4* 3′UTR across ten mammalian species. A phylogenetic tree depicts the evolutionary distance of *PLIN4* 3′UTRs across these species (**Figure 4a**). The *PLIN4* 3′UTR has undergone significant change from mouse to primate and furthermore that change is ongoing even among recently diverged primates such as neandertal and human. **Figure 4b** shows an alignment of the last 100 bases of the *PLIN4* 3′UTR. The reference nucleotide for all sequences except mouse, neandertal and human is a C at the rs8887 position. The mouse has no orthologous sequence for this segment and neandertal and human sequence have a G or an A/G nucleotide, respectively, suggesting the recent emergence of the 522 MRE.

To investigate if the recent rs8887 SNP is undergoing selection across human populations, we utilized the fixation index (FST) statistic, which measures the divergence of alleles across populations [29]. The frequency of a particular allele in populations can vary over time and is influenced by such forces as genetic drift and natural selection and FST values can be used to approximate these influences. FST statistics among total and population subdivisions of HapMap PHASE III data were obtained with the SNP@Evolution web tool (**Table 5**) [30]. The total FST value for rs8887 was 0.132 indicating a moderate level of differentiation between populations, suggesting rs8887 is undergoing drift.

**Table 5:** FST values for rs88887 among HapMap Phase III data.

Populations	FST
**ASN**	0.01678
**EUR**	0.00258
**AFR**	0.03766
**AME**	0.08720
**AEAA**	0.13228

FST: differentiation among populations.

ASN: samples of Asian,CHB, CHD, and JPT.

CHB: Han Chinese in Beijing, China.

CHD: Chinese in Metropolitan Denver, Colorado.

JPT: Japanese in Tokyo, Japan.

EUR: samples of European, CEU and TSI.

CEU: Utah residents with Northern and Western European ancestry.

TSI: Toscans in Italy.

AFR: samples of African, YRI, ASW, LWK, and MKK.

YRI:  Yoruba in Ibadan, Nigeria.

ASW: African ancestry in Southwest USA.

LWK: Luhya in Webuye, Kenya.

MKK: Maasai in Kinyawa, Kenya.

AME: samples of American,GIH and MEX.

GIH: Gujarati Indians in Houston, Texas.

MEX: Mexican ancestry in Los Angeles, California.

AEAA: ASN, EUR, AFR and AME.

## Discussion

We report novel associations between SNPs in human *PLIN4* and obesity related phenotypes. We also have identified a series of gene by diet interactions modulating these traits. Of particular interest rs8887 associates with a constellation of anthropometric traits which were modulated through interaction with dietary PUFA. *In silico* analysis of the *PLIN4* mRNA sequence predicted the minor A allele of rs8887 generates a novel seed site for miR-522 and our *ex vivo* luciferase data indicated that miR-522 reduced PLIN4 protein levels 20% via the *PLIN4* 3′UTR target site created by the rs8887 A allele. Importantly, single point mutations in MRE seed sites have shown the ability to reduce or abolish miR-mediated repression [31].

When 3′UTR sequences drift over the course of evolution, they are continuously exposed to potential matches with co-expressed miRs. While conservation signal is often used to predict functional MREs, it has been determined that a conservation signal above background for MREs of the most recent mammalian specific miRNA families was unlikely due to the relatively short time between the emergence of these miRs and the occurrence of new MREs within 3′UTRs [9]. This suggests that for some of the more recent MREs to emerge, there has not been sufficient time for environment to determine which sites are beneficial, neutral or detrimental with respect to the genome. It is likely that some primate 3′UTRs have been, and are, subject to drift via the appearance of new genetic variants, resulting in loss or gain of miR-target interactions which may have potential for phenotypic modulation [20]. However, to date, there are few known examples of genetic variation in miR-target sites contributing to phenotypic variation [32].

C19MC is thought to be a product of an AluJ/AluS insertion into chromosome 19 during an early stage of primate evolution suggesting a role for miR-522 in higher development and phenotypic plasticity [26]. It is likely that miR-522 is important for development given its temporal expression in placenta and fetal tissues, however its role in adipocytes is unknown. Our phylogenetic analysis indicates that variation at the rs8887 position resulting in the *PLIN4* miR-522 MRE is specific to humans and likely undergoing drift. We hypothesize that binding between rs8887 and mir-522 results in suboptimal expression of *PLIN4*, thereby contributing to the elevation in anthropometrics observed in our association analyses. A layer of complexity in controlling expression of the *PTEN* oncogene has been described as an interaction between a microRNA and pseudogene *PTENP1* [33]. We find no evidence for a *PLIN4* pseudogene in the human genome which strengthens the implications of the miR-522-PLIN4 interaction we describe here.

If the appearance of variation at the rs8887 position is recent to human, it is tempting to speculate that the genesis of the rs8887 minor A allele may have contributed to the phenotypic diversification distinguishing humans from other primates as it is thought that the gain, or loss, of genetic regulatory mechanisms are critical for the evolutionary process [34]. That this interaction may have contributed to the evolution of the human brain is food for thought [35].

Our association data indicate for rs8887 minor allele carriers that elevated intake of PUFA n3 results in decreasing anthropometrics compared to non-carriers. Due to what little is known of *PLIN4* regulation, it is difficult to propose a mechanism by which the miR-522 rs8887 interaction together with PUFA n3 could modulate anthropometrics. It is likely that PUFA n3 alters *PLIN4* expression through PPAR mediated pathways [28]. Furthermore, studies in model organisms have demonstrated anti-obesity effects of PUFA n3 which are thought to mediate their effects by modulating the activity of various transcription factors important to lipid metabolism [36]. It may be miR-522 is dysregulated in the obese and thus contributes to the dysregulation of adipogenic pathways as suggested for another C19MC member, miR-519d [37]. Alternatively, miR-522 may be modulated by environmental factors which influence *PLIN4* through rs8887 as suggested for several other miRs [38]. If in addition to regulating *PLIN4* expression PUFA n3 down-regulates miR-522, we would expect increasing PUFA n3 intake to have a more dramatic effect in reducing weight for subjects carrying the minor allele compared to non-carriers. Specifically, if the miR-522 *PLIN4* interaction is absent in those homozygous for the G allele, reducing miR-522 activity through increasing PUFA n3 will have no additional effect on increasing *PLIN4* expression, and therefore no added contribution to weight loss. FOS subjects have on average less PUFA n3 intake than GOLDN subjects (**Table 1**). In addition, our associations for baseline anthropometrics are more significant in FOS, while p-values for interaction with PUFA n3 are less significant compared to GOLDN values (**Tables 3** **,** **4**). The reduced levels of PUFA n3 intake in FOS subjects possibly bias associations toward significance of main effects, while biasing against significance for interaction with PUFA n3. Identifying the function(s) of miR-522 and the conditions that induce its activation and repression will help clarify its role in mammalian development and as a potential modulator of obesity phenotypes.

We can only speculate how lower expression of PLIN4 contributes to obesity-related phenotypes. For the related PLIN1, one study demonstrated that obesity and high lipolysis rates are independently associated with lower PLIN1 protein levels in women, whereas another demonstrated reduced levels of both *PLIN1* mRNA and protein in obese compared to non-obese subjects [39], [40]. Conversely, the *Plin1^−/−^* mouse is characterized by a lean phenotype. These data suggest the role and regulation of the PAT gene family in human obesity may be different than in model organisms.

In addition to our findings with rs8887, the rs884164 variant showed significant interaction with PUFA n3 modulating anthropometric and lipid traits. The rs884164 variant was predicted to fall in an NFkB motif. NFkB acts downstream of *TNFa* signaling and is thought to contribute to the pro-inflammatory response observed obese individuals [41]. In addition to the activation of other pro-inflammatory cytokines, which can lead to disruptions in insulin signaling, NFkB is thought to up-regulate lipogenic factors and down-regulate adipogenic factors thereby increasing serum FFAs and further contributing to the insulin resistance and CVD associated with obesity [42]. Laurencikiene *et al* demonstrated *in vitro* that lipolysis was abolished upon the inactivation of NFkB in human adipocytes. Moreover, NFkB was shown to elevate *PLIN1* and Hormone Sensitive Lipase (*HSL*) expression during lipolytic stimulation [43]. It has been demonstrated that PUFA n3 can modulate the expression levels of NFkB target genes [44]. PUFA n3s were shown to decrease levels of NFkB target genes by limiting the translocation of NFkB subunits from the cytoplasm to the nucleus [45]. However, the extent through which NFkB affects energy storage and expenditure is not well characterized in humans. If and how rs884164 may modulate PLIN4 response to PUFA through NFkB remains to be determined.

Several variants at the *PLIN4* locus are estimated to explain a small portion of the variance observed in anthropometric traits from main effects and by the interaction of these variants with PUFA intake (**Tables 3** **,** **4**). These estimates do not appear to account for a large amount of phenotypic variability. However, as with the case of *FTO* and BMI, common variants modulating anthropometric traits often explain only a small amount of the observed phenotypic variation [46]. It is likely that variation at *PLIN4* is yet another contributor to the complex nature of obesity and its associated comorbidities.

A potential limitation to this study, given the hypothesis driven nature of our analyses and the correlation between traits examined, is a lack of adjusting our results for multiple-tests. Furthermore, there was some heterogeneity in the levels of statistical significance between the FOS and GOLDN populations, which may be due to the larger sample size of FOS or may be explained in part by differing PUFA n3 intakes between populations. However, we are confident in our findings given that the direction of the effect in both populations was the same, that our meta-analysis demonstrated overall significance and that the rs8887 functional data support our conclusions. The type and quantity of fat in the diet are an important factor in determining risk for obesity. To this end, a variety of dietary recommendations are suggested for obesity prevention and therapy. A concern regarding these recommendations is accounting for possible inconsistencies introduced by other factors affecting the desired outcomes. The data presented here offer an example of this occurrence in that subjects carrying the rs8887 minor allele are potentially more sensitive to lowering their previously elevated anthropometrics by increasing their PUFA n3 intake. This work may help enable health professionals to better tailor an effective weight-loss regimen based on a patients DNA profile.

## Materials and Methods

### Statistical analysis

A dominant model was applied to all SNPs classifying homozygotes for the major allele in one group, and carriers and homozygotes of the minor allele in another. Multivariate linear regression was used for association analyses in the GOLDN and FOS populations. To reduce variability that might obscure potential findings, multiple covariates were incorporated into our regression model, including age, sex, alcohol and tobacco smoking status, physical activity (GOLDN only), hormone use and diabetes, cholesterol and hypertension medications. To adjust for familial relationships among subjects in both populations, the lme *kinship* procedure was used in R, which allows the specification of the full correlation structure within pedigrees into the regression equation with random effects. To determine gene by diet interactions a SNP*dietary term was introduced into the regression equation. These tests were adjusted for total energy intake by adding total energy to the model, in addition to those mentioned above. A p value<.05 was considered significant. Response variables that did not maintain a normal distribution were log transformed to fit the normal distribution.

Meta-analyses were performed with the software package Meta-Analysis helper (METAL) (www.sph.umich.edu/csg/abecasis/metal) which combines results from two or more individual studies. We used meta analysis to weight the effect size of each study by its sample size and combining Z statistics to determine an overall level of significance.

### 3′UTR Luciferase Reporter Assays

The Expand High Fidelity PCR kit (Roche) was used to amplify the *PLIN4* 3′UTR sequence using gDNA from subjects homozygous for either the rs8887 G or A allele. Primers were designed to amplify a 560 nucleotide sequence including the last 460 bases of the PLIN4 3′UTR and 100 bases of downstream genomic sequence. Included in the primers were the restriction enzyme sites XhoI for the forward primer ( ***AACTCGAGCTGTAGGAGCCTGCAAG*** ) and NotI for the reverse ( ***AGCGGCCGCGACTATAAATGGTTTTTTAATGAAAAAAGAAATCACT*** ). These PCR products where cloned into the multiple cloning site of the PSICHeCK2 reporter vector downstream of the Renilla luciferase coding sequence.

COS7 cells, plated into 12-well plates (Costar), were co-transfected with 1 µg of the pmiR-LucPLIN4-G or pmiR-LucPLIN4-A luciferase reporter vectors and 40 nM mi*RIDIAN* miR-522 mimic or an equal concentration of a non-targeting control mimic sequence (Dharmacon) using the Lipofectamine 2000 Reagent (Invitrogen). Luciferase activity was measured using the Dual-Glo Luciferase Assay System (Promega). *Renilla* luciferase activity was normalized to the corresponding firefly luciferase activity and plotted as a percentage of the control (cells co-transfected with the corresponding concentration of control mimic). This experiment was performed in triplicate wells of a 12-well plate and repeated at least three times.

### Study design and subjects FOS

The design and methods of the Framingham Offspring Study, which was initiated in 1971, have been reported [47]. Blood samples for DNA were collected between 1987 and 1991. Anthropometric, lipid and dietary intake variables were recorded for subjects who participated in the fifth and sixth examination visits. Variables used in this study consist of mean values calculated from these two exams, with the exception of fasting insulin and HOMA measurements that were available only for subjects participating in exam 5. Dietary intake was determined with a semi-quantitative food frequency questionare [48]. Intakes of PUFA (n3 and n6) where calculated for each subject and used in our analyses as continuous variables. The Institutional Review Boards (IRB) for Human Research at Boston University and Tufts University/New England Medical Center approved the protocol. All participants provided written informed consent.

### Anthropometric and Biochemical determinations for FOS

Briefly, weight was measured with the individual dressed in an examining gown and wearing no shoes. The BMI was calculated as weight in kilograms divided by the square of height in meters. Fasting glucose, plasma lipids, and lipoproteins were measured as previously described [49]. To analyze SAT and VAT variables, data from the Framingham Heart Study Multidetector Computed Tomography Study, a population-based sub-study of the community-based Framingham Heart Study Offspring and Third-Generation Study cohorts were used [21].

### Study design and subjects GOLDN

Study protocol approval was obtained from the Human Studies Committee of Institutional Review Board at the University of Minnesota, University of Utah, and Tufts Medical Center. All participants provided written informed consent. The detailed methodology and design of the GOLDN study has been described previously [17], [50]. Briefly, GOLDN is part of the Program for the Genetic Interactions Network and is funded by the NIH. Participants were recruited from pedigrees from two genetically homogenous National Heart, Lung, and Blood Institute Family Heart Study field centers in Minnesota and Utah, both predominately white populations. There were 1086 subjects with complete phenotype, dietary and genotype data. Dietary intake was estimated by use of the Dietary Health Questionnaire (DHQ) which consists of 124 food items and includes both portion size and dietary supplement questions [51].

### Anthropometric and biochemical determinations for GOLDN

Blood samples were drawn after fasting overnight. Anthropometrics and blood collection, plasma separation and processing, and biochemical lipid measurements, including triglycerides and HDL cholesterol, have been described previously [52]. Fasting plasma insulin was determined by the Human Insulin Specific RIA kit (Linco Research). Fasting plasma glucose was measured using a hexokinase-mediated reaction on a Hitachi 911 (Roche Diagnostics).

### SNP selection and genotyping

To identify common SNPs in the human *PLIN4* locus, we searched the HapMap database for polymorphic alleles with a minor allele frequency ≥5%. The *PLIN4* locus was defined as 5000 bp upstream of the predicted start codon, and 2000 bp downstream from the mRNA endpoint, a region spanning approximately 25.1 Kb. Thirteen SNPs were identified using these criteria.

We chose seven of these SNPs for genotyping; two promoter (rs884164 and rs1609717), one exonic missense (rs7250947), one 3′UTR (rs8887) and three intronic (rs8102428, rs892158, and rs11673616). Analysis of the HapMap CEU population with the Haploview program determined rs892158 (*rs7260518 and rs10406797*), rs7250947 (rs*8102428 and rs884164*), rs11673616 (*rs4991027*) and rs1609717 (*rs4807598*) to be tagSNPs capturing variants in LD with an r^2^>0.8, predicting coverage over eleven of thirteen SNPs in the region [53].

DNA was isolated from blood samples using DNA blood Midi kits (Qiagen, Hilden, Germany) according to the vendor's recommended protocol. Ready-made 5′ nucleic allelic discrimination assays were available from Applied Biosystems for *PLIN4* SNPs rs8887, rs11673616, rs892158, rs8102428, and rs884164. We used the Applied Biosystems Custom Assay design web tool to generate functional assays for SNPs rs1609717, and rs7250947 (appliedbiosystems.com). We genotyped *PLIN4* SNPs using the Taqman assays listed above on the ABIPrism 7900HT Sequence Detection System (Applied Biosystems). Standard laboratory practices were used to ensure accuracy of the data.

### Cell culture & RNA isolation

HepG2, COS-7 and HEK-293T (obtained from American Type Tissue Collection) cells were maintained in Dulbecco's Modified Eagle Medium (DMEM) containing 10% FBS and 2% penicillin-streptomycin. RNA was extracted from approximately 6×10^6^ cells using Trizol reagent. Total RNA was reverse transcribed using the RT2 miRNA First Strand kit (SABiosciences) and miRNA quantified using primers specific for human miR-522 (SABiosciences qPCR assay) and values were normalized to the housekeeping gene SNORD38b. All experiments were performed in triplicate. Trizol whole cell lysates from 3×10^6^ human pre- and mature adipocytes were purchased from Zen-Bio. Total RNA was purified using the miRNeasy kit (Qiagen) and miRNA was quantified using the protocol above.

### Phylogenetic analysis

Nucleotide sequences for human, neandertal, chimpanzee, gorilla, orangutan, lemur, wild boar, cow, dog and mouse were downloaded from reference assemblies available at NCBI and aligned with ClustalW. The evolutionary history of PLIN4 was inferred using the Maximum Parsimony (MP) method. The bootstrap consensus tree inferred from 10000 replicates is taken to represent the evolutionary history of the taxa analyzed. Branches corresponding to partitions reproduced in less than 50% bootstrap replicates are collapsed. The percentage of replicate trees in which the associated taxa clustered together in the bootstrap test (10000 replicates) are shown next to the branches. The MP tree was obtained using the Close-Neighbor-Interchange algorithm with search level 3 [2], [3] in which the initial trees were obtained with the random addition of sequences (100 replicates) [54]. The analysis involved 10 nucleotide sequences. There were a total of 2447 positions in the final dataset. Evolutionary analyses were conducted in MEGA4. Total and population FST statistics for HapMap populations where estimated using the SNP@Evolution webtool which implements the FST calculations described in Akey et al [29].

## Supporting Information

Table S1 **Additional significant associations observed for baseline and interaction association analyses.** Results of meta-analysis in FOS and GOLDN performed using a Dominant Model. P-values for anthropometrics were adjusted for sex, age, smoking, physical activity (GOLDN only), alcohol use, diabetes, beta-blockers, calories from fat, PUFA n3 and n6, and estrogen and menopausal status (FOS only) in women. Lipid and glucose p-values where also adjusted for BMI and cholesterol medications. Gene by diet interaction for meta-analysis of FOS and GOLDN. Interactions between *PLIN4* variants and dietary PUFA n3 and n6 were included in a multivariate regression model as continuous variables. Values in the top table are for main effect analyses, and the bottom table for interactions analyses.
(DOC)

## References

[1] Ogden C (2006). Prevalence of Overweight and Obesity in the United States,
1999–2004.. JAMA: The Journal of the American Medical Association.

[2] Fontaine KR, Redden DT, Wang C, Westfall AO, Allison DB (2003). Years of life lost due to obesity.. JAMA.

[3] Koutsari C (2006). Thematic review series: Patient-Oriented Research. Free fatty
acid metabolism in human obesity.. The Journal of Lipid Research.

[4] Vanherpen N, Schrauwenhinderling V (2008). Lipid accumulation in non-adipose tissue and
lipotoxicity.. Physiol Behav.

[5] Brasaemle DL (2007). Thematic review series: Adipocyte Biology. The perilipin family
of structural lipid droplet proteins: stabilization of lipid droplets and
control of lipolysis.. J Lipid Res.

[6] Marinescu VD, Kohane IS, Riva A (2005). MAPPER: a search engine for the computational identification of
putative transcription factor binding sites in multiple
genomes.. BMC Bioinformatics.

[7] Martinez-Botas J, Anderson JB, Tessier D, Lapillonne A, Chang BH (2000). Absence of perilipin results in leanness and reverses obesity in
Lepr(db/db) mice.. Nature Genetics.

[8] Tansey JT, Sztalryd C, Gruia-Gray J, Roush DL, Zee JV (2001). Perilipin ablation results in a lean mouse with aberrant
adipocyte lipolysis, enhanced leptin production, and resistance to
diet-induced obesity.. Proceedings of the National Academy of Sciences of the United States of
America.

[9] Friedman RC, Farh KK, Burge CB, Bartel DP (2009). Most mammalian mRNAs are conserved targets of
microRNAs.. Genome Res.

[10] Bell CG, Walley AJ, Froguel P (2005). The genetics of human obesity.. Nat Rev Genet.

[11] Lai C (2006). Dietary Intake of n-6 Fatty Acids Modulates Effect of
Apolipoprotein A5 Gene on Plasma Fasting Triglycerides, Remnant Lipoprotein
Concentrations, and Lipoprotein Particle Size: The Framingham Heart
Study.. Circulation.

[12] Corella D, Ordovas J (2005). SINGLE NUCLEOTIDE POLYMORPHISMS THAT INFLUENCE LIPID METABOLISM:
Interaction with Dietary Factors.. Annu Rev Nutr.

[13] Schoonjans K, Staels B, Auwerx J (1996). Role of the peroxisome proliferator-activated receptor (PPAR) in
mediating the effects of fibrates and fatty acids on gene
expression.. Journal of lipid research.

[14] Qi L, Tai E, Tan C, Shen H, Chew S (2005). Intragenic linkage disequilibrium structure of the human
perilipin gene (PLIN) and haplotype association with increased obesity risk
in a multiethnic Asian population.. J Mol Med.

[15] Qi L, Shen H, Larson I, Schaefer EJ, Greenberg AS (2004). Gender-specific association of a perilipin gene haplotype with
obesity risk in a white population.. Obes Res.

[16] Qi L, Corella D, Sorlí JV, Portolés O, Shen H (2004). Genetic variation at the perilipin (PLIN) locus is associated
with obesity-related phenotypes in White women.. Clin Genet.

[17] Corella D (2006). Perilipin Gene Variation Determines Higher Susceptibility to
Insulin Resistance in Asian Women When Consuming a High-Saturated Fat,
Low-Carbohydrate Diet.. Diabetes Care.

[18] Kang E (2006). The 11482G>A Polymorphism in the Perilipin Gene Is Associated
With Weight Gain With Rosiglitazone Treatment in Type 2
Diabetes.. Diabetes Care.

[19] Stark A, Brennecke J, Bushati N, Russell RB, Cohen SM (2005). Animal MicroRNAs confer robustness to gene expression and have a
significant impact on 3′UTR evolution.. Cell.

[20] Saunders MA, Liang H, Li WH (2007). Human polymorphism at microRNAs and microRNA target
sites.. Proc Natl Acad Sci U S A.

[21] Fox C, Massaro J, Hoffmann U, Pou K, Maurovich-Horvat P (2007). Abdominal Visceral and Subcutaneous Adipose Tissue Compartments:
Association With Metabolic Risk Factors in the Framingham Heart
Study.. Circulation.

[22] Grimson A, Farh KK, Johnston WK, Garrett-Engele P, Lim LP (2007). MicroRNA targeting specificity in mammals: determinants beyond
seed pairing.. Mol Cell.

[23] Betel D, Wilson M, Gabow A, Marks DS, Sander C (2008). The microRNA.org resource: targets and
expression.. Nucleic Acids Res.

[24] Marinescu VD, Kohane IS, Riva A (2005). The MAPPER database: a multi-genome catalog of putative
transcription factor binding sites.. Nucleic Acids Res.

[25] Bentwich I, Avniel A, Karov Y, Aharonov R, Gilad S (2005). Identification of hundreds of conserved and nonconserved human
microRNAs.. Nat Genet.

[26] Zhang R, Wang YQ, Su B (2008). Molecular evolution of a primate-specific microRNA
family.. Mol Biol Evol.

[27] Landgraf P, Rusu M, Sheridan R, Sewer A, Iovino N (2007). A mammalian microRNA expression atlas based on small RNA library
sequencing.. Cell.

[28] Dalen KT, Schoonjans K, Ulven SM, Weedon-Fekjaer MS, Bentzen TG (2004). Adipose tissue expression of the lipid droplet-associating
proteins S3-12 and perilipin is controlled by peroxisome
proliferator-activated receptor-gamma.. Diabetes.

[29] Akey JM, Zhang G, Zhang K, Jin L, Shriver MD (2002). Interrogating a high-density SNP map for signatures of natural
selection.. Genome Res.

[30] Cheng F, Chen W, Richards E, Deng L, Zeng C (2009). SNP@Evolution: a hierarchical database of positive selection on
the human genome.. BMC Evol Biol.

[31] Brennecke J, Stark A, Russell RB, Cohen SM (2005). Principles of microRNA-target recognition.. PLoS Biol.

[32] Sethupathy P, Collins FS (2008). MicroRNA target site polymorphisms and human
disease.. Trends Genet.

[33] Poliseno L, Salmena L, Zhang J, Carver B, Haveman WJ (2010). A coding-independent function of gene and pseudogene mRNAs
regulates tumour biology.. Nature.

[34] King MC, Wilson AC (1975). Evolution at two levels in humans and
chimpanzees.. Science.

[35] Cunnane SC, Crawford MA (2003). Survival of the fattest: fat babies were the key to evolution of
the large human brain.. Comp Biochem Physiol A Mol Integr Physiol.

[36] Sampath H, Ntambi J (2005). POLYUNSATURATED FATTY ACID REGULATION OF GENES OF LIPID
METABOLISM.. Annu Rev Nutr.

[37] Martinelli R, Nardelli C, Pilone V, Buonomo T, Liguori R (2010). miR-519d overexpression is associated with human
obesity.. Obesity.

[38] Rayner KJ, Suarez Y, Davalos A, Parathath S, Fitzgerald ML (2010). MiR-33 contributes to the regulation of cholesterol
homeostasis.. Science.

[39] Mottagui-Tabar S, Ryden M, Lofgren P, Faulds G, Hoffstedt J (2003). Evidence for an important role of perilipin in the regulation of
human adipocyte lipolysis.. Diabetologia.

[40] Wang Y, Sullivan S, Trujillo M, Lee MJ, Schneider SH (2003). Perilipin expression in human adipose tissues: effects of severe
obesity, gender, and depot.. Obes Res.

[41] Juge-Aubry CE, Henrichot E, Meier CA (2005). Adipose tissue: a regulator of inflammation.. Best Pract Res Clin Endocrinol Metab.

[42] Ruan H, Hacohen N, Golub TR, Van Parijs L, Lodish HF (2002). Tumor necrosis factor-alpha suppresses adipocyte-specific genes
and activates expression of preadipocyte genes in 3T3-L1 adipocytes: nuclear
factor-kappaB activation by TNF-alpha is obligatory.. Diabetes.

[43] Laurencikiene J, van Harmelen V, Arvidsson Nordström E, Dicker A, Blomqvist L (2007). NF-kappaB is important for TNF-alpha-induced lipolysis in human
adipocytes.. J Lipid Res.

[44] Camandola S, Leonarduzzi G, Musso T, Varesio L, Carini R (1996). Nuclear factor kB is activated by arachidonic acid but not by
eicosapentaenoic acid.. Biochem Biophys Res Commun.

[45] Zhao Y, Joshi-Barve S, Barve S, Chen LH (2004). Eicosapentaenoic acid prevents LPS-induced TNF-alpha expression
by preventing NF-kappaB activation.. J Am Coll Nutr.

[46] Frayling TM, Timpson NJ, Weedon MN, Zeggini E, Freathy RM (2007). A common variant in the FTO gene is associated with body mass
index and predisposes to childhood and adult obesity.. Science.

[47] Feinleib M, Kannel WB, Garrison RJ, McNamara PM, Castelli WP (1975). The Framingham Offspring Study. Design and preliminary
data.. Prev Med.

[48] Rimm EB, Giovannucci EL, Stampfer MJ, Colditz GA, Litin LB (1992). Reproducibility and validity of an expanded self-administered
semiquantitative food frequency questionnaire among male health
professionals.. Am J Epidemiol.

[49] Corella D, Lai CQ, Demissie S, Cupples LA, Manning AK (2007). APOA5 gene variation modulates the effects of dietary fat intake
on body mass index and obesity risk in the Framingham Heart
Study.. J Mol Med.

[50] Shen J, Arnett DK, Peacock JM, Parnell LD, Kraja A (2007). Interleukin1beta genetic polymorphisms interact with
polyunsaturated fatty acids to modulate risk of the metabolic
syndrome.. J Nutr.

[51] Thompson FE, Subar AF, Brown CC, Smith AF, Sharbaugh CO (2002). Cognitive research enhances accuracy of food frequency
questionnaire reports: results of an experimental validation
study.. J Am Diet Assoc.

[52] Lai CQ, Demissie S, Cupples LA, Zhu Y, Adiconis X (2004). Influence of the APOA5 locus on plasma triglyceride, lipoprotein
subclasses, and CVD risk in the Framingham Heart Study.. J Lipid Res.

[53] Barrett JC, Fry B, Maller J, Daly MJ (2005). Haploview: analysis and visualization of LD and haplotype
maps.. Bioinformatics.

[54] Tamura K, Dudley J, Nei M, Kumar S (2007). MEGA4: Molecular Evolutionary Genetics Analysis (MEGA) software
version 4.0.. Mol Biol Evol.

